# Low immunity against vaccine preventable diseases in Turkish HIV cohort

**DOI:** 10.3906/sag-2102-14

**Published:** 2021-10-21

**Authors:** Aslıhan CANDEVİR, Ferit KUŞCU, Figen YILDIRIM, Süheyla KÖMÜR, Gönül Çiçek ŞENTÜRK, Ayşe Seza İNAL, Fatma ESER, Salih ÇETİNER, Behice KURTARAN, Yeşim TAŞOVA

**Affiliations:** 1 Department of Infectious Diseases and Clinical Microbiology, Faculty of Medicine, Çukurova University, Adana Turkey; 2 Department of Infectious Diseases and Clinical Microbiology, University of Medical Sciences, Antalya Training and Research Hospital, Ankara Turkey; 3 Department of Infectious Diseases and Clinical Microbiology, University of Medical Sciences, Dışkapı Training and Research Hospital, Ankara Turkey; 4 Department of Immunology, Faculty of Medicine, Çukurova University, Adana Turkey

**Keywords:** HIV, immunity, measles, mumps, rubella, tetanus

## Abstract

**Background/aim:**

HIV infection increase the risk of serious disease resulting from common vaccine-preventable infections. Vaccinations are particularly important for HIV infected adults. We aimed to investigate the immunity rates against measles, mumps, rubella, hepatitis A, B, and tetanus in newly diagnosed HIV patients.

**Materials and methods:**

Patients who admitted to outpatient clinics of three centers with newly diagnosed HIV infection, between 1 January 2015 and 31 June 2017 were included. Measles, mumps, rubella, varicella zoster virus, hepatitis A, hepatitis B, and tetanus antibody levels were measured by commercial diagnostic kits. Demographical and laboratory data of the patients were recorded.

**Results:**

Five hundred and twenty-three patients were enrolled in the study. Of the patients 87% were male (n = 455) and the mean age was 38 ± 13 years. Serology was available for measles 74.2% (388/523), mumps 73.8% (386/523), rubella 77.8% (407/523), hepatitis A 88.5% (463/523), hepatitis B 97.7% (511/523), tetanus 8.6% (45/523), and VZV 79.9% (418/523). Seropositivity was 82% for measles, 75.6% for mumps, 92.1% for rubella. Of the patients whom all three of the components of the MMR vaccine was tested, 37.7% (127/337) were susceptible at least one and needed the vaccine. Mean age was lower in patients who are nonimmune to measles and mumps (p = 0.008). Younger patients were also nonimmune for hepatitis A, while older patients were nonimmune for hepatitis B.

**Conclusion:**

In our study we found that rates of nonimmunity can increase up to one third of the patients even though there is a national vaccination program. Nonimmune individuals should be detected and vaccinated in line with recent guidelines and response should be monitored because of the possibility of impaired immunity and possible suboptimal response. National campaigns can be launched for adult immunization and physicians should be aware of the importance of adult immunization.

## 1. Introduction

HIV has infected more than 75 million people worldwide and an estimated 37 million people are now living with the virus. HIV infection is one of the main causes of morbidity and mortality worldwide [1]. HIV infection increases the risk of serious disease resulting from common vaccine-preventable infections and affects the quality, quantity, and longevity of immune responses against natural infection or vaccination [2].

Vaccinations are particularly important for HIV infected adults. Due to impaired host defenses. HIV infected persons have both an increased risk and severity of vaccine-preventable infections [3]. Suboptimal response to vaccines has been observed in patients with advanced HIV infection [4]. Life expectancy and quality of life have markedly increased in HIV-positive individuals since the introduction of antiretroviral therapy (ART) [5]. Thus, the likelihood of HIV-positive individuals being in contact with vaccine-preventable infections in occupational, social, and travel exposures has increased substantially. At the same time, studies from other countries have demonstrated low frequency of seropositivity against measles, mumps, and rubella (MMR) or Varicella Zoster virus (VZV) in HIV-positive patients, especially in young adults [2,6–11]. 

To our knowledge, this is the first study investigating the status of seropositivity to the vaccine-preventable diseases in the population living with HIV in Turkey. We aimed to investigate the immunity rates against measles, mumps, rubella, VZV, hepatitis A, B, and tetanus in newly diagnosed HIV patients in order to protect these patients from diseases that can be prevented easily and cost effectively by just a vaccine. 

## 2. Material and methods

In the study, HIV/AIDS patient records were investigated retrospectively in Çukurova University Medical School and University of Medical Sciences, Antalya and Dışkapı Training and Research Hospitals’ Infectious Diseases clinics between January 2015 and December 2017. Patients’ data were extracted from electronic records and patient charts and anonymized. Demographic data, CD4 (+) lymphocyte count and HIV RNA levels on diagnosis, immunity against measles, mumps, rubella, tetanus, hepatitis A, and B were recorded. Ethical committee approval was obtained from Çukurova University Ethical Board (2021/49). 

HAV IgG, anti-HBs, anti-HBc IgG, Rubella IgG tests were performed with electro-chemiluminescence immunoassay (ECLIA) using the Cobas E601 analyzer (Roche Diagnostics). VZV IgG, measles IgG, mumps IgG tests were performed with the micro-ELISA method (Viro-Immun Labor-Diagnostika GmbH, Oberursel, Germany) in Çukurova University and Antalya Training and Resarch Hospitals. In Dışkapı Training and Research Hospital available serologic test kits were used at the time patients admitted. Tetanus IgG levels were measured using a *Clostridium tetani *5S IgG ELISA kit (Novatec Immundiagnostica GmBH, Germany) at only one center, Çukurova University Hospital. 

Statistical analyses were performed using SPSS v. 20.0 software package. The results were presented as mean ± standard deviation or median (min-max), for continuous variables and percentage (%) was used for categorical variables. Normality was checked using the Kolmogorov–Smirnov test for each continuous variable. When comparing groups, the student’s t-test was used for normally distributed data and the Mann–Whitney U test for not normally distributed data. Comparison of the categorical variables between the groups was done using the Chi square and Fischer’s Exact test. 

## 3. Results

Newly diagnosed 523 patients were included to the study between January 2015 and December 2017 from three centers. Contributions of patients from the centers were as follows; Çukurova University Medical School Hospital 52.2% (n = 273), University of Medical Sciences, Antalya Training and Research Hospitals 31% (n = 162) and Dışkapı Training and Resarch Hospital 16.8% (n = 88). Of the patients 87% were male (n = 455) and the mean age was 38 ± 13 years. Vast majority of the patients were Turkish citizens while 19 (3.6%) were migrants. Mean CD4 (+) lymphocyte count and HIV RNA levels were 418 ± 272 /mm^3 ^and 733,396 ± 2,645,569 copy/mL at the time of diagnosis, respectively. Variations of some characteristics in the patients according to different hospitals were shown in Table 1.

**Table 1:** Variations of some characteristics in the patients according to different hospitals.

Hospital	N (%)	Age years(Mean ± SD)	Male sexN (%)	Being migrant N (%)	CD4 /mm3(Mean ± SD)	HIV RNA copy/mL(Mean ± SD)
CUMS	273 (52.2)	34.5 ± 11.6	243 (89.0)	6 (2.2)	431 ± 270	610692 ± 1802165
ATRH	162 (31.0)	40.5 ± 13.7	136 (84.0)	11 (6.8)	387 ± 266	575,940 ± 1,892,162
DYBTRH	88 (16.8)	42.8 ± 13.2	76 (86.4)	2 (2.3)	431 ± 283	1,524,340 ± 5,292,797
p value	-	<0.0001*	0.310	0.035**	0.249	0.025***

CUMS: Çukurova University Medical School, AT RH: Antalya Training and Research Hospital, DYBTRH: Dışkapı Training and Research Hospital.

Serology was available for measles 74.2% (388/523), mumps 73.8% (386/523), rubella 77.8% (407/523), hepatitis A 88.5% (463/523), hepatitis B 97.7% (511/523), tetanus 8.6% (45/523), and VZV 79.9% (418/523). Seropositivity was 82% for measles, 75.6% for mumps, 92.1% for rubella and 37.7% (127/337) of the patients were susceptible to at least one component of the vaccine and needed MMR vaccine. Immunity status of these diseases among HIV positive patients and relationship to age, sex, CD4 count, and HIV RNA is showed in Table 2. 

**Table 2:** Immunity status of various diseases among HIV positive patients according to age, sex, being an immigrant, CD4 count, and HIV RNA.

Diseases	Immune status	N (%)	Age, years(Mean ± SD)	Male sexN (%)	Being migrantN (%)	CD4, /mm3(Mean ± SD)	HIV RNA, copy/mL(Mean ± SD)
Measles	NI	70 (18)	33.1 ± 11.5	64 (91.4)	1 (7.1)	461 ± 268	645,400 ± 2,007,672
	I	318 (82)	37.4 ± 12.6	278 (87.4)	13 (92.9)	426 ± 282	672,723 ± 2,470,072
	P		0.008	0.419	0.480	0.239	0.198
Mumps	NI	94 (24.4)	33.9 ± 11.4	84 (89.4)	3 (21.4)	505 ± 291	1,445,558 ± 152,375
	I	292 (75.6)	37.4 ± 13.0	254 (87.0)	11 (78.6)	421 ± 269	2,414,227 ± 145,583
	p		0.023	0.595	1.00	0.008	0.153
Rubella	NI	32 (7.9)	39.9 ± 14.5	28 (87.5)	0 (0)	459 ± 265	217,545 ± 41,112
	I	375 (92.1)	36.2 ± 12.3	330 (88.0)	15 (100)	424 ± 267	2,961,056 ± 157,156
	p		0.200	1.00	0.619	0.514	0.401
Varicella	NI	66 (15.8)	34.9 ± 11.2	58 (87.9)	4 (25.0)	457 ± 268	1,187,788 ± 4,051,754
	I	352 (84.2)	37.1 ± 12.8	309 (87.8)	12 (75.0)	416 ± 277	707,267 ± 2,474,292
	p		0.227	0.983	0.296	0.194	0.589
Hepatitis A	NI	78 (16.8)	31.1 ± 12.2	69 (88.5)	4 (23.5)	428 ± 262	1,038,975 ± 2,501,138
	I	385 (83.2)	38.4 ± 12.6	336 (87.3)	13(76.5)	437 ± 263	485,262 ± 1,573,092
	p		<0.001	0.772	0.505	0.687	0.252
Hepatitis B	NI	302 (60.0)	38.7 ± 11.9	257 (85.1)	11 (57.9)	400 ± 270	676,682 ± 2,081965
	I	201 (40.0)	36.0 ± 15.4	180 (89.6)	8 (42.1)	441 ± 217	699,046 ± 2,708,563
	p		<0.001	0.7147	1.00	0.032	0.976
Tetanus	NI	27 (60)	40.6 ± 14.3	23 (85.2)	0 (0)	348 ± 217	92,788 ± 101,630
	I	18 (40)	33.3 ± 11.7	15 (83.3)	1(100)	405 ± 214 ±	1,719,250 ± 3,811,376
	p		0.112	1.00	1.00	0.331	0.610

NI: Nonimmune. I: Immune.

Mean age was lower in patients who are nonimmune to measles (33.1 ± vs. 37.4 ± 12.6. p = 0.008) and mumps (33.9 ± 11.4 vs. 37.4 ± 13.0, p = 0.023). Younger patients were also nonimmune for hepatitis A (p < 0.001), while older patients were nonimmune for hepatitis B (p < 0.001). There was no difference in CD4 counts, except for mumps (nonimmune; 505 ± 291 vs. immune; 421 ± 269, p = 0.008). 

Seropositivity of patients to mentioned diseases according to age groups are showed in Figure. There was a relationship between seropositivity and age groups for hepatitis A (p < 0.0001), B (p < 0.0001), and measles (p = 0.014). Hepatitis B antibody positivity was highest in 27–36 age group. It increased after decreasing in the latter two decades in the ≥ 48 age group parallel with anti-HBc positivity (p < 0.0001) in this decade. Hepatitis A, mumps, and measles seropositivity increased with the increasing age groups. There was a decline in tetanus immunity by increasing age groups, but it was not statistically significant (p = 0.188). Increase of the seropositivity to mumps and varicella wasn’t significant either (p values are 0.133 and 0.187 respectively) (Figure). 

**Figure 1:**
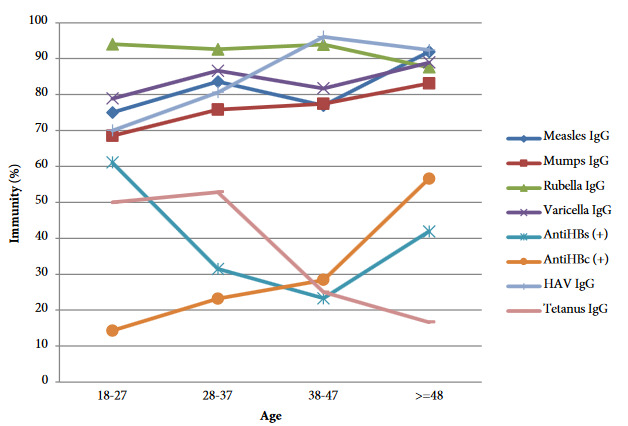
Immunity rates of patients to vaccine-preventable diseases according to age groups.

## 4. Discussion

HIV-infected persons are at an increased risk for various infections. Some infections are more common in this population while some infections cause more serious clinical conditions. Acquisition of hepatitis B virus (HBV) infection is common because of the shared routes of transmission, and progression rate to significant liver disease (cirrhosis, hepatocellular carcinoma) is higher compared with HIV-uninfected persons. However, HIV infection does not predict a more severe course for hepatitis A, although prolonged viremia has been described [12,13]. HIV patients also have a higher incidence of varicella zoster virus (VZV) infections and related deaths [14,15]. Measles infections can be particularly severe, with life-threatening infections; in contrast mumps and rubella clinic is usually like the immune competent hosts [16]. Similarly, there is no evidence of poorer outcomes of tetanus in HIV positives and recommendations are the same with the negatives [17]. Vaccination is a key component to ensure the health of all persons living with HIV by preventing infectious complications and determination of the immune status to vaccine preventable diseases will be the first step achieving this. 

There is a lack of published national data regarding the seropositivity of vaccine preventable diseases in adult population in Turkey. Nearly all studies in adults investigated the immune status of the healthcare workers (HCWs) and high seropositivity rates are seen in these studies [18–23]. Alp et al. in the largest study reported that, of the 1255 HCW’s 94% were immune to measles, 97% to rubella, 90% to mumps, and 98% to varicella. Except one, in the other studies measles, mumps, rubella, and varicella seroprevalence rates were also high and between 81.6%–99.7%, 80%–99.7%, 85.5%–98.8%, and 71%–99.7% respectively. In one study, tetanus, and diphtheria seroprevalences were reported as 93.5% and 60.8% and they showed the decreasing positivity by age. This study also mentioned hepatitis B antibody rate as 84.1% of which 71.5% was vaccinated [20]. In another study investigating hepatitis epidemiology HBs antibody positivity was found to be 36.12% [24]. In contrast two population-based studies show lower rates of seropositivity probably healthcare personnel’s being more aware of vaccine preventable diseases [25,26]. In the cross-sectional study of Emek et al., seroprevalence of measles in the whole study population of 1250 was 82.2%. The seroprevalence of measles was found lower than expected and was particularly low in subjects aged 30 years of age. In the other study, antibody levels against measles conferred protection in 98% of patients, 65% of the patients had no protection for diphtheria, 69% had no protection for tetanus, and 90% of the patients had no protection for pertussis. Only 1.3% of the study population had seropositivity against three of the diseases. In a population-based household survey investigating varicella zoster serology that 2136 healthy persons participated 94.3% of individuals were seropositive for varicella virus [27]. In the largest and recent population-based study from Turkey investigating hepatitis, TURHEP study, anti-HBs positivity was identified 31.9% in of which sampling was performed from 23 cites in 2009–2010 living in urban and rural areas by two-stage stratified method, consistent with the before mentioned studies [28]. In a study among asylum seekers in the Netherlands, overall, seroprotection was 84% (ranged between 54%–100% in different races) for hepatitis A and 27% for hepatitis B (anti-HBs; 8%–42%) [29]. Also, in a study from İstanbul hepatitis A seroprevalence was 69% in those 20–25 years old [30]. And tetanus seropositivity was 98% (86%–100%) in the study investigating asylum seekers from Syria, Iran, Iraq, Afghanistan, Eritrea, and Ethiopia, living in the Netherlands.

The need for vaccination varies by place and time. A study of 700 HIV-infected adults in Austria noted the rate of seronegative 8.4% for measles, 33.4% for mumps, and 18.8% for rubella; overall, almost half were lacking immunity to one of the components and required MMR vaccination [11]. Additionally, a study from Spain reported that nearly 30% were seronegative to at least one component of the MMR vaccine [10]. In these studies, they found immigrants at the greatest need for vaccination. We didn’t find increased need for vaccination for the immigrants possibly because of the low number and heterogenicity. BHIVA guidelines suggest screening HIV-infected adults for measles IgG regardless of history of childhood vaccination [31]. Also screening for rubella IgG is recommended among HIV-positive women of childbearing age with unknown status. In our study susceptibility was 18% for measles, 24.4% for mumps, 7.9% for rubella, and totally 37.7% required MMR vaccine. We see that immunity rates are lower than the available literature and under the herd immunity thresholds but due to the lack of the population-based studies low rates of seroprevalence cannot be linked to HIV [32]. Nevertheless, there are similar results of low seropositivity in HIV positive community and it should be considered that impaired immunity of HIV positive individuals may cause poor vaccine responses [11]. Also, studies investigating the reasons and risk factors of low immunity, such as vaccine refusal or change in vaccination programs should be conducted. Currently, guidelines recommend two doses of MMR among nonimmune persons with a CD4 count ≥ 200 cells/mm^3^ [33].

Most guidelines recommend determination of the VZV immune status. Among the general population, waning of immunity has been noted by 8 years post-vaccination, but there are no current studies regarding durability among HIV-infected adults [34]. Many HIV-infected adults were shown to have immunity, with 95% of US HIV-infected adults and with a 98% in the UK was seropositive to VZV although there is geographic variation [35,36]. Seroprevalence was lower in our series but similar seroprevalences were reported from healthy populations and we didn’t see any waning by age. BHIVA, EACS, and French guidelines recommend varicella vaccination among nonimmune persons with a CD4 count ≥ 200 cells/mm (14%) [33]. Therefore, there is no clinical data on the immunogenicity of varicella vaccination among seronegative HIV-infected adolescents or adults.

In a study from France, 81.8% of HIV patients were considered immunized against HAV virus following natural infection or vaccination [37]. In another study from the UK of 200 patients of whom 43.5% were born out of the country, seropositivity rate was 79.5% (34.5% recalled vaccination) [36]. Hepatitis A immunity of 83.2% is similar with the studies among HIV positive and population-based studies. Also, the direct correlation of seropositivity with age in our study is consistent with the studies demonstrating lower seropositivity among younger adults [30]. 

In a study investigating migrants 97 patients (39%, 95% CI; 33.2–45.8) were considered cured (anti-HBs plus anti-HBc, no HBs Ag), 25 patients (10%, 95% CI; 6.4–13.9) were protected (anti-HBs, no anti-HBc and no HBsAg) and 64 patients (25.8%, 95% CI; 20.3–31.3) had only anti-HBc antibodies [38]. In the study of Molton et al., 76/200 (38.0%) showed detectable anti-HBc antibody, consistent with a past infection [36]. In our study, hepatitis B antibody positivity was 60% higher than literature. Seropositivity was highest in 17–25 age group consistent with the vaccination coverage started in 1998 and the second highest age group was > 45 age group which was parallel to the anti HBc positivity consistent with the prior infection. 

Mullaert et al. also investigated the tetanus seroprotection and the rate was 70.8% overall (95% CI; 65.0–76.3), higher in women (78.8%, vs. 58.2%) [38]. Tetanus serology was available from 711 patients in a study from Austria and 361 (51%) tested positive [39]. Tetanus seropositivity of 60% in our study is consistent with the available literature and takes attention to the need of vaccination in this patient group. Guidelines recommend giving a Td booster every 10 years, especially among those at risk for exposure. Among those over 50 years, shortening the interval for booster doses to every 5 years is suggested [17]. Seroprotection after boosters can be investigated at this immune suppressed group for widening this recommendation for the people living with HIV as we see low protection levels. 

HIV infection increases the risk of serious, sometimes life-threatening disease resulting from common vaccine-preventable infections. As we showed in our study that rates of susceptibility can increase nearly to one third of the patients even though there is a national vaccination program, susceptible individuals should be detected and vaccinated in line with recent guidelines and response should be monitored because of the possibility of impaired immunity and possible suboptimal response. National coverage rates are also important due to herd immunity and possible effect on susceptible populations, besides in order to increase the immunity rates; national campaigns should be thrown for adult immunization and physicians should be aware of the importance of adult immunization. 

## Informed consent

Ethical committee approval was obtained from Cukurova University Ethical Board (2021/49) and anonymized data was used. No informed consent was needed.

## References

[ref1] Overbaugh J Phillips A Buchbinder S. 2015 HIV infection Nature Reviews Disease Primers 1 15035 15035 10.1038/nrdp.2015.35 27188527

[ref2] Molton J Smith C Chaytor S Maple P Brown K 2010 Seroprevalence of common vaccine-preventable viral infections in HIV-positive adults Journal of Infection 61 73 80 10.1016/j.jinf.2010.04.004 20403382

[ref3] Crum-Cianflone NF Wallace MR 2014 Vaccination in HIV-infected adults AIDS Patient Care and STDs 28 397 410 25029589 10.1089/apc.2014.0121 PMC4117268

[ref4] Kernéis S Launay O Turbelin C Batteux F Hanslik T 2014 Long-term immune responses to vaccination in HIV-infected patients: a systematic review and meta-analysis Clinical Infectious Diseases 58 1130 1139 24415637 10.1093/cid/cit937 PMC4761378

[ref5] Ehren K Hertenstein C Kümmerle T Vehreschild JJ Fischer J 2014 Causes of death in HIV-infected patients from the Cologne-Bonn cohort Infection 42 135 140 24081925 10.1007/s15010-013-0535-7

[ref6] Kemper CA Gangar M Arias G Kane C Deresinski SC 1998 The prevalence of measles antibody in human immunodeficiency virus-infected patients in northern California Journal of Infectious Disesases 178 1177 1180 10.1086/515679 9806055

[ref7] Kemper CA Zolopa AR Hamilton JR Fenstersheib M Bhatia G 1992 Prevalence of measles antibodies in adults with HIV infection: possible risk factors of measles seronegativity AIDS 6 1321 1325 1472336 10.1097/00002030-199211000-00013

[ref8] Choudhury SA Hatcher F Berthaud V Ladson G Hills E 2008 Immunity to measles in pregnant mothers and in cord blood of their infants: impact of HIV status and mother’s place of birth Journal of the National Medical Association 100 1445 1449 19110913 10.1016/s0027-9684(15)31545-5

[ref9] Lambert D Dramé M Rouger C Brodard V Nguyen Y 2015 High prevalence of measles seronegativity in adults with HIV infection born in the era of measles vaccination in Northern France AIDS 29 241 243 25486416 10.1097/QAD.0000000000000549

[ref10] Llenas-García J Rubio R Hernando A Arrazola P Pulido F 2013 Do HIV-positive adult immigrants need to be screened for measles-mumps-rubella and varicella zoster virus immunization? AIDS Care 25 980 989 23244745 10.1080/09540121.2012.748881

[ref11] Grabmeier-Pfistershammer K Poeppl W Herkner H Touzeau-Roemer V Huschka E 2014 High need for MMR vaccination in HIV infected adults in Austria Vaccine 32 6020 6023 25203449 10.1016/j.vaccine.2014.07.114

[ref12] Thio CL Seaberg EC Skolasky R Phair J Visscher B hepatitis 2002 B virus, and risk of liver-related mortality in the Multicenter Cohort Study (MACS) Multicenter AIDS Cohort Study. HIV-1 360 1921 1926 10.1016/s0140-6736(02)11913-1 12493258

[ref13] Costa-Mattioli M Allavena C Poirier AS Billaudel S Raffi F 2002 Prolonged hepatitis A infection in an HIV-1 seropositive patient Journal of Medical Virology 68 7 11 12210424 10.1002/jmv.10163

[ref14] Veenstra J Van Praag RM Krol A Wertheim van Dillen PM Weigel HM 1996 Complications of varicella zoster virus reactivation in HIV-infected homosexual men AIDS 10 393 399 8728043 10.1097/00002030-199604000-00007

[ref15] Gebo KA Kalyani R Moore RD Polydefkis MJ 2005 The incidence of, risk factors for, and sequelae of herpes zoster among HIV patients in the highly active antiretroviral therapy era Journal of Acquired Immune Deficiency Syndromes 40 169 174 16186734 10.1097/01.qai.0000178408.62675.b0

[ref16] Kaplan LJ Daum RS Smaron M McCarthy CA 1992 Severe measles in immunocompromised patients JAMA 267 1237 1241 1538561

[ref17] Crum-Cianflone NF Sullivan E 2017 Vaccinations for the HIV-infected adult: a review of the current recommendations part I. Infectious Diseases and Therapy 6 303 331 28779442 10.1007/s40121-017-0166-x PMC5595780

[ref18] Alp E Cevahir F Gökahmetoglu S Demiraslan H Doganay M. 2012 Prevaccination screening of health-care workers for immunity to measles, rubella, mumps, and varicella in a developing country: What do we save? Journal of Infection and Public Health 5 127 132 22541258 10.1016/j.jiph.2011.11.003

[ref19] Aypak C Bayram Y Eren H Altunsoy A Berktaş M. 2012 Susceptibility to measles, rubella, mumps, and varicella-zoster viruses among healthcare workers Journal of Nippon Medical School 79 453 458 23291844 10.1272/jnms.79.453

[ref20] Cılız N Gazi H Ecemiş T Şenol Ş Akçalı S 2013 Sağlık çalışanlarında Kızamık 26 26 30

[ref21] Çalık Ş Tosun S Tuncel Başoğlu M Sayın S. 2017 The Investigation of Seroprevalence of Measles, Rubella, Mumps, and Varicella in Medical Students FLORA 22 73 77

[ref22] Dilli D Dallar Y Önde U Doğan F Yağcı S. 2008 Ergenlerde kızamık, kızamıkçık, kabakulak ve su çiçeği prevalansı Çocuk Dergisi 8 172 178

[ref23] Köse Ş Mandiracıoğlu A Egemen A 2006 Erişkinlerde kızamık antikor seropozitifliğinin değerlendirilmesi Ege Tıp Dergisi 45 93 95

[ref24] Guclu E Ogutlu A Karabay O. A 2016 Study on the age-related changes in hepatitis B and C virus serology The Eurasian Journal of Medicine 48 37 41 27026763 10.5152/eurasianjmed.2015.85 PMC4792495

[ref25] Emek M Islek D Atasoylu G Ozbek OA Ceylan A 2017 Association between seroprevalence of measles and various social determinants in the year following a measles outbreak in Turkey Public Health 147 51 58 28404496 10.1016/j.puhe.2017.01.026

[ref26] Tanriover MD Soyler C Ascioglu S Cankurtaran M Unal S 2014 Low seroprevalence of diphtheria, tetanus and pertussis in ambulatory adult patients: the need for lifelong vaccination European Journal of Internal Medicine 25 528 532 24814432 10.1016/j.ejim.2014.04.010

[ref27] Kose S Mandiracioglu A Senger SS Ulu Y Cavdar G 2013 Seroprevalence of varicella-zoster virus in the prevaccine era: a population-based study in Izmir, Turkey J Infection and Public Health 6 115 119 10.1016/j.jiph.2012.10.003 23537824

[ref28] Tozun N Ozdogan O Cakaloglu Y Idilman R Karasu Z 2015 Seroprevalence of hepatitis B and C virus infections and risk factors in Turkey: a fieldwork TURHEP study Clinical Microbiology and Infection 21 1020 1026 26163105 10.1016/j.cmi.2015.06.028

[ref29] Freidl GS Tostmann A Curvers M Ruijs WLM Smits G 2016 Immunity against measles, mumps, rubella, varicella, diphtheria, tetanus, polio, hepatitis A and hepatitis B among adult asylum seekers in the Netherlands Vaccine 36 1664 1672 10.1016/j.vaccine.2018.01.079 29454516

[ref30] Ceran N Yüksel Kocdogan F Mert D Erdem I Dede B 2012 Hepatitis A seroprevalence in children and young adults in Istanbul, Turkey: seroprevalence change and associated factors Journal of Viral Hepatitis 19 72 76 22187947 10.1111/j.1365-2893.2011.01454.x

[ref31] Palumbo P Hoyt L Demasio K Oleske J Connor E 1992 Population-based study of measles and measles immunization in human immunodeficiency virus-infected children The Pediatric Infectious Diseases Journal 11 1008 1014 10.1097/00006454-199211120-00004 1461690

[ref32] Fine PE 1993 Herd immunity: history, theory, practice Epidemiologic Reviews 15 265 302 8174658 10.1093/oxfordjournals.epirev.a036121

[ref33] Frésard A Gagneux-Brunon A Lucht F Botelho-Nevers E Launay O 2016 Immunization of HIV-infected adult patients - French recommendations Human Vaccines & Immunotherapeutics 12 2729 2741 27409293 10.1080/21645515.2016.1207013 PMC5137523

[ref34] Morrison VA Johnson GR Schmader KE Levin MJ Zhang JH 2015 Shingles prevention study group. Long-term persistence of zoster vaccine efficacy Clinical Infectious Diseases 60 900 909 25416754 10.1093/cid/ciu918 PMC4357816

[ref35] Crum-Cianflone NF Sullivan E 2017 Vaccinations for the HIV-infected adult: a review of the current recommendations, part II Infectious Diseases and Therapy 6 333 361 28780736 10.1007/s40121-017-0165-y PMC5595779

[ref36] Molton J Smith C Chaytor S Maple P Brown K 2010 Seroprevalence of common vaccine-preventable viral infections in HIV-positive adults Journal of Infection 61 73 80 10.1016/j.jinf.2010.04.004 20403382

[ref37] Valour F Cotte L Voirin N Godinot M Ader F 2014 Vaccination coverage against hepatitis A and B viruses, Streptococcus pneumoniae, seasonal flu, and A(H1N1) 2009 pandemic influenza in HIV-infected patients Vaccine 32 4558 4564 24951870 10.1016/j.vaccine.2014.06.015

[ref38] Mullaert J Abgrall S Lele N Batteux F Slama LB 2015 Diphtheria, tetanus, poliomyelitis, yellow fever and hepatitis B seroprevalence among HIV1-infected migrants. Results from the ANRS VIHVO vaccine sub-study Vaccine 33 4938 4944 26209841 10.1016/j.vaccine.2015.07.036

[ref39] Grabmeier-Pfistershammer K Herkner H Touzeau-Roemer V Rieger A Burgmann H 2015 Low tetanus, diphtheria and acellular pertussis (Tdap) vaccination coverage among HIV infected individuals in Austria Vaccine 33 3929 3932 26102535 10.1016/j.vaccine.2015.06.056

